# Antibacterial Properties of Tebipenem Pivoxil Tablet, a New Oral Carbapenem Preparation against a Variety of Pathogenic Bacteria *in Vitro* and *in Vivo*

**DOI:** 10.3390/molecules21010062

**Published:** 2016-01-06

**Authors:** Qi Yao, Jingkun Wang, Tao Cui, Zhi Yang, Mei Su, Peiyue Zhao, Hong Yan, Yi Zhan, Hongbo Yang

**Affiliations:** 1 Department of Pharmacology, Yunnan Institute of Materia Medica, Kunming 650111, China; yaoqi313@126.com (Q.Y.); m_ssu@sina.com (M.S.); hong_yyan@163.com (H.Y.); yi_zhan91@tom.com (Y.Z.); 2 Department of GLP, Yunnan Institute of Materia Medica, Kunming 650111, China; jkun_wang@126.com (J.W.); hb_yyang@sina.com (H.Y.); 3 Department of Scientific Management, Yunnan Institute of Materia Medica, Kunming 650111, China; c_ttao@126.com; 4 Department of Chemical Pharmaceutics, Yunnan Institute of Materia Medica, Kunming 650111, China; pyy_zhao@sohu.com

**Keywords:** tebipenem pivoxil, pathogenic bacteria, MIC, 100% MLD, bacterial sepsis

## Abstract

Aims: To systemically investigate the *in vitro* and *in vivo* antibacterial properties of tebipenem pivoxil tablet. In addition, acute toxicity of this preparation was also studied. Methods: *In vitro*, minimum inhibitory concentration (MIC) or minimal inhibitory concentration (MBC) were determined by using the serial 2-fold broth or agar dilution methods. Further, cumulative MIC inhibition curves were then made to assess the antibacterial effects of the drug at various concentrations. *In vivo*, minimum lethal dose (MLD) in combination with maximum tolerance dose (MTD) was used to measure the acute toxicity of the tebipenem pivoxil tablet in mice. After that, sepsis mouse models challenged with *Escherichia coli*, *Staphylococcus aureus*, *Pseudomonas aeruginosa*, and *Klebsiella pneumoniae*, respectively, were established to evaluate the anti-infective effect of this preparation. Results: The MIC_90_ values of tebipenem pivoxil against Gram-positive bacteria such as methicillin-sensitive *Staphylococcus aureus* (MSSA), methicillin-resistant *Staphylococcus aureus* (MRSA), methicillin-sensitive *Staphylococcus epidermidis* (MSSE), methicillin-resistant *Staphylococcus epidermidis* (MRSE), *Pyogenic streptococcus*, and *Enterococcus faecalis* were ≤0.125, 16, 0.5, 8, ≤0.125, and 32 μg/mL, respectively. Correspondingly, the MIC_90_ values of tebipenem pivoxil against *Escherichia coli*, *Klebsiella* *pneumoniae*, *Enterobacter aerogenes*, *Haemophilus influenzae*, *Pseudomonas aeruginosa*, and *Acinetobacter baumannii* were 1, 0.5, ≤0.125, 0.25, 64, 64 μg/mL, respectively. The MBC values of tebipenem pivoxil against *Escherichia coli*, *Staphylococcus aureus*, *Klebsiella pneumoniae* were 0.016–2, 0.063–32, 0.031–32 μg/mL, respectively. The acute toxicity study showed that the MLD of the tebipenem pivoxil tablet was 4.00 g/kg and the MTD was 3.40 g/kg in mice. In all the sepsis mouse models, the simultaneous administration of the tebipenem pivoxil tablets significantly reduced mortality of the sepsis-model mice as compared with the control. Furthermore, the survival rate in the tebipenem pivoxil tablet group was remarkably higher than that in the meropenem group in all the sepsis mouse models tested. In the sepsis model challenged with *Staphylococcus aureus* ATCC29213, *Escherichia coli* ATCC25922, *Pseudomonas aeruginosa* ATCC27853, and *Pseudomonas aeruginosa* clinical strain, respectively, tebipenem pivoxil tablet (100 mg/kg) displayed a better protective effect than tebipenem pivoxil granules (100 mg/kg). Conclusions: In summary, tebipenem pivoxil displays an excellent antibacterial activity against a variety of pathogenic bacteria *in vitro*. Importantly, tebipenem pivoxil tablet significantly protects the sepsis mice challenged with various pathogenic bacteria, which may provide a potential approach to treating bacterial sepsis in clinic.

## 1. Introduction

Tebipenem pivoxil, belonging to the carbapenem class, was firstly developed by Pfizer Inc. (New York, NY, USA) and its granule preparation, a product of Meiji Inc., (Tokyo, Japan), was approved and then listed in April 2009. Like the β-lactam antibiotics, tebipenem pivoxil binds penicillin-binding protein (PBP), thereby inhibiting cell wall synthesis. A nitrogen heterocyclic group at the C3 side chain in the chemical structure forms a prodrug by interacting with C2 carboxylic acids, significantly elevating oral absorption, which plays a key role in a better antibacterial activity of tebipenem pivoxil compared with most other β-lactam antibiotics.

Tebipenem pivoxil has a broad antibacterial spectrum [1,2]. Briefly, it has a stronger antibacterial activity than penicillin or cephalosporin series compounds against majority of Gram-positive and -negative bacteria such as methicillin-resistant *Saphylococcus aureus* (MRSA), methicillin-resistant *Staphylococcus epidermidis* (MRSE), *Pyogenic streptococcus*, *Enterococcus faecalis*, *Escherichia coli*, *Klebsiella pneumoniae*, *Enterobacter aerogenes*, *Pseudomonas aeruginosa*, and so on, in most clinical isolates [1]. Compared with other carbapenem antibiotics, tebipenem pivoxil shows more efficient antibacterial activity, especially for infections in children mainly due to penicillin-resistant *Streptococcus pneumoniae* (PRSP), *Erythromycin-resistant Streptococcus pneumoniae* (MRSP) and *Haemophilus influenzae* (Hib).

In Japan, tebipenem pivoxil is commonly applied in the clinic in the therapy of otolaryngological and upper respiratory tract infections [3,4,5], and its therapeutic efficiency is significantly higher than that of imipenem, amoxicillin, levofloxacin, *etc.* So far, the effect of tebipenem pivoxil in bacterial sepsis has not been reported yet.

Now, clinical data show that sepsis morbidity caused by Gram-positive bacteria is increasing gradually. Among these, the morbidity caused by *Staphylococcus aureus* is a lead cause. *Saphylococcus aureus* is a major pathogen responsible for infections after burns, acute liver failure, acute nephritis, and so on. Sepsis caused by *Saphylococcus aureus* is often frequently accompanied by Gram-negative bacteria such as *Pseudomonas aeruginosa* and *Klebsiella pneumoniae*, which seriously threatens the lives of patients by synergy [6].

In the present study the antibacterial properties of the tebipenem pivoxil were firstly investigated against a variety of pathogenic bacteria *in vitro*. This tebipenem pivoxil tablet was then used in the treatment of sepsis mouse models challenged with common pathogenic bacteria. In view of this, we hoped to clarify the clinical application potential of tebipenem pivoxil in the treatment of some infectious diseases such as bacterial sepsis.

## 2. Results

### 2.1. Tebipenem Pivoxil Displays Excellent Antibacterial Activity against a Variety of Pathogenic Bacteria in Vitro

**Figure 1:**
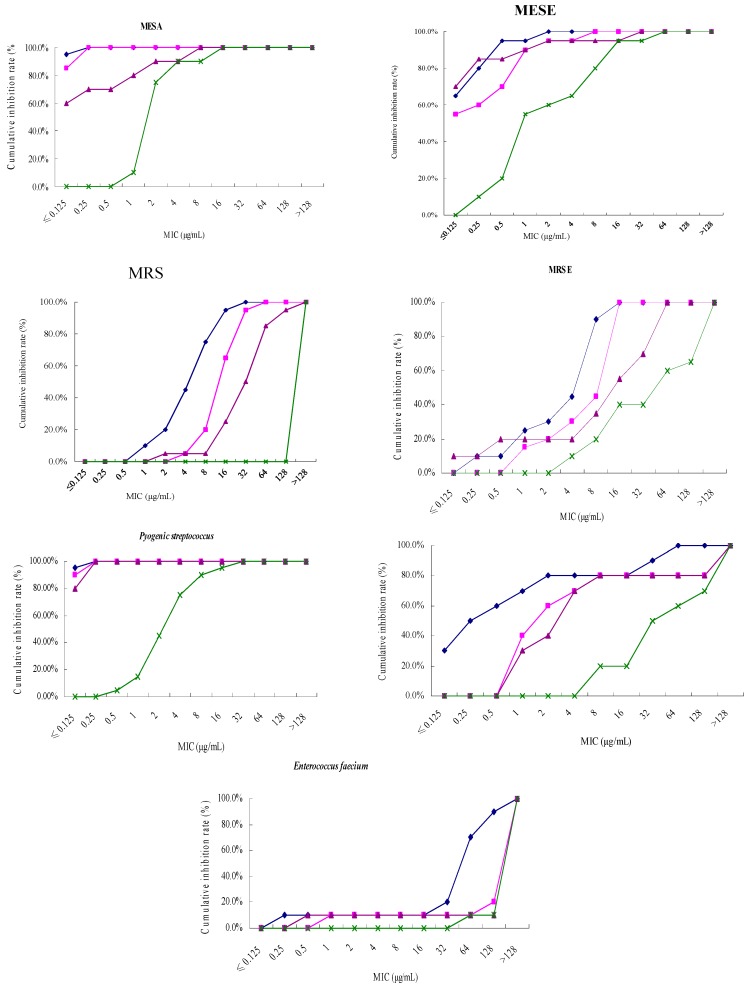
Cumulative inhibition curves of tebipenem pivoxil and other antibiotics against gram-positive bacteria tested. ◆, Tebipenem Pivoxil; ■, Meropenem; ▲, Imipenem and cilastatin; ×, Ceftriaxone.

The MIC_90_ values of tebipenem pivoxil against MSSA, MRSA, MSSE, MRSE were ≤0.125, 16, 0.5, 8 μg/mL, lower than meropenem (0.25, 32, 1, 16 μg/mL), imipenem and cilastatin (2, 128, 1, 64 μg/mL), and ceftriaxone (4, >128, 16, >128 μg/mL). The antibacterial activity of tebipenem pivoxil against *Pyogenic streptococcus* was similar to that of meropenem (both MIC_90_ ≤ 0.125 μg/mL) and stronger than those of imipenem and cilastatin (MIC_90_ = 0.25 μg/mL) and ceftriaxone (MIC_90_ = 8 μg/mL). The MIC_90_ values of tebipenem pivoxil against *Enterococcus faecalis* and *Enterococcus faecium* were 32 and 128 μg/mL, respectively, lower than meropenem (>128 and >128 μg/mL), imipenem and cilastatin (>128 and >128 μg/mL), and ceftriaxone (>128 and >128 μg/mL) (Table 1 and Figure 1).

**Table 1:** MICs of tebipenem pivoxil and other antibiotics against tested Gram-positive bacteria.

Strain	MIC_50_/MIC_90_ (μg/mL)			
	Tebipenem Pivoxil	Meropenem	Imipenem and Cilastatin	Ceftriaxone
MSSA	≤0.125/0.125	≤0.125/0.25	≤0.125/2	2/4
MRSA	8/16	16/32	32/128	>128/>128
MSSE	≤0.125/0.5	≤0.125/1	≤0.125/1	1/16
MRSE	8/8	16/16	16/64	64/>128
*Enterococcus faecalis*	0.25/32	2/>128	4/>128	32/>128
*Enterococcus faecium*	64/128	>128/>128	>128/>128	>128/>128
*Pyogenic streptococcus*	≤0.125/≤0.125	≤0.125/≤0.125	≤0.125/0.25	4/8

**Figure 2:**
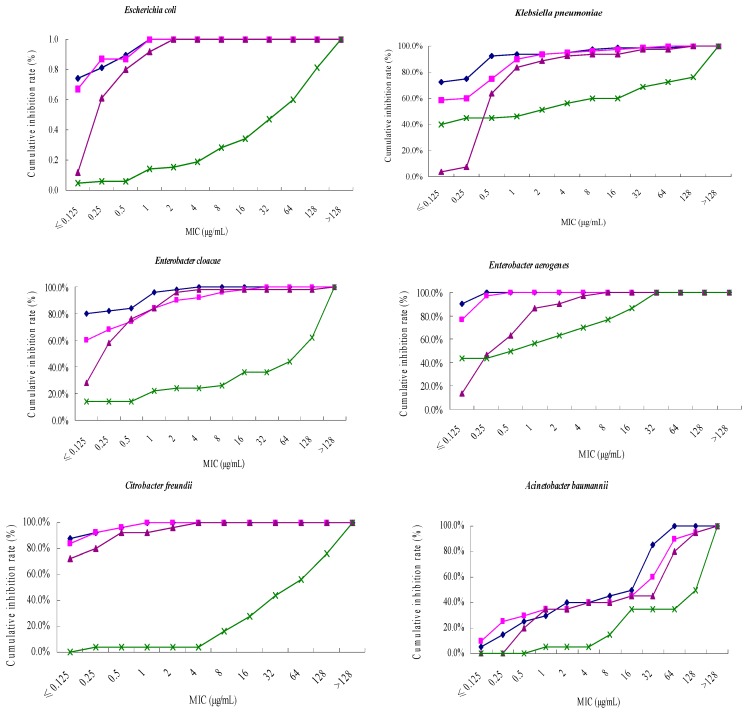

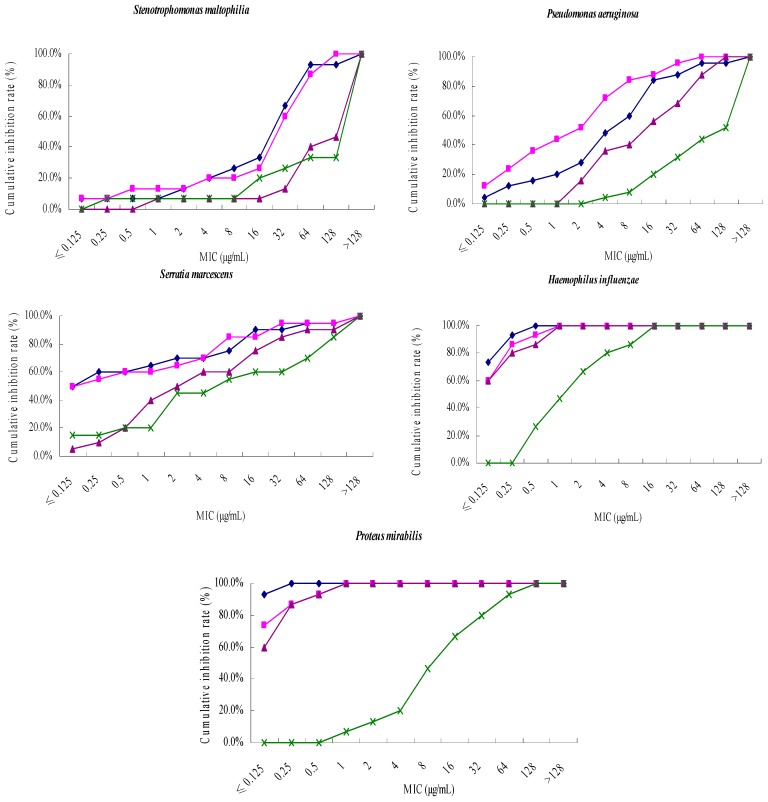
Cumulative inhibition curves of tebipenem pivoxil and other antibiotics against gram-negative bacteria tested. ◆, Tebipenem Pivoxil; ■, Meropenem; ▲, Imipenem and cilastatin; ×, Ceftriaxone.

Among all the Gram-negative bacteria tested, tebipenem pivoxil had a significant antibacterial activity against *Escherichia coli*, with a MIC_90_ value of 1 μg/mL, which was as same as meropenem, imipenem and cilastatin (MIC_90_ = 1 μg/mL), and significantly higher than ceftriaxone (MIC_90_ > 128 μg/mL). The antibacterial activity of tebipenem pivoxil against *Klebsiella pneumoniae*, *Enterobacter aerogenes*, *Haemophilus influenzae* (MIC_90_ = 0.5, ≤0.125, 0.25 μg/mL) was better than that of meropenem (MIC_90_ = 1, 0.25, 0.5 μg/mL), imipenem and cilastatin (MIC_90_ = 4, 2, 1 μg/mL), and ceftriaxone (MIC_90_ = >128, 32, 16 μg/mL). For *Enterobacter cloacae*, *Proteus mirabilis*, *Citrobacter freundii* and *Serratia marcescens*, the MIC_90_ values of tebipenem pivoxil were 1, ≤0.125, 0.25, 16 μg/mL, respectively, lower than meropenem (2, 0.5, 0.5, 32 μg/mL), imipenem and cilastatin (2, 0.5, 0.5, 64 μg/mL), and ceftriaxone (>128, 64, >128, >128 μg/mL). The MIC_90_ values of tebipenem pivoxil against *Stenotrophomonas maltophilia* and *Acinetobacter baumannii* were 64 μg/mL, higher than meropenem (32, 32 μg/mL), and lower than imipenem and cilastatin (128, 128 μg/mL), and ceftriaxone (>128, >128 μg/mL), respectively. The MIC_90_ of tebipenem pivoxil against *Pseudomonas aeruginosa* was 64 μg/mL, higher than meropenem (32 μg/mL), lower than imipenem and cilastatin (128 μg/mL), and ceftriaxone (>128 μg/mL). The antibacterial activity of tebipenem pivoxil against *Haemophilus influenzae* and *Proteus mirabilis* (MIC_90_ = 0.25, ≤0.125 μg/mL) was stronger than that of meropenem (MIC_90_ = 0.5, 0.5 μg/mL), imipenem and cilastatin (MIC_90_ = 1, 0.5 μg/mL), and ceftriaxone (MIC_90_ = 16, 64 μg/mL) (Table 2 and Figure 2).

**Table 2:** MICs of tebipenem pivoxil and other antibiotics against the tested Gram-negative bacteria.

Strain	MIC_50_/MIC_90_ (μg/mL)			
	Tebipenem Pivoxil	Meropenem	Imipenem and Cilastatin	Ceftriaxone
*Escherichia coli*	≤0.125/1	≤0.125/1	0. 25/1	64/>128
*Klebsiella pneumoniae*	≤0.125/0.5	≤0.125/1	0.5/4	2/>128
*Enterobacter cloacae*	≤0.125/1	≤0.125/2	0.25/2	128/>128
*Enterobacter aerogenes*	≤0.125/≤0.125	≤0.125/0.25	0.5/2	0.5/32
*Citrobacter freundii*	≤0.125/0.25	≤0.125/0.25	≤0.125/0.5	64/>128
*Acinetobacter baumannii*	16/64	32/64	64/128	128/>128
*Stenotrophomonas maltophilia*	32/64	32/128	>128/>128	>128/>128
*Pseudomonas aeruginosa*	8/64	2/32	16/128	128/>128
*Serratia marcescens*	≤0.125/16	≤0.125/32	2/64	8/>128
*Haemophilus influenzae*	≤0.125/0.25	≤0.125/0.5	≤0.125/1	2/16
*Proteus mirabilis*	≤0.125/≤0.125	≤0.125/0.5	≤0.125/0.5	16/64

The MBC values of tebipenem pivoxil were determined in 10 *Escherichia coli*, 10 *Staphylococcus epidermidis*, and 10 *Klebsiella pneumoniae*. The results showed that the MBC values of tebipenem pivoxil against *Escherichia coli*, *Staphylococcus aureus*, *Klebsiella pneumoniae* were 0.016–2, 0.063–32, 0.031–32 μg/mL respectively. The MBC values were 1–8 times, 2–8 times, 2–8 times the MIC value against *Escherichia coli*, *Staphylococcus aureus*, *Klebsiella pneumoniae*, respectively, suggesting a strong bactericidal activity of tebipenem pivoxil against *Escherichia coli*, *Staphylococcus aureus*, *Klebsiella pneumoniae* (Table 3).

**Table 3:** MBC/MIC values of tebipenem pivoxil in *Escherichia coli*, *Staphylococcus aureus*, and *Klebsiella pneumonia*.

Strain	MBC/MIC (μg/mL)
*Escherichia coli*	
ATCC25922	0.031/0.016
Clinical strain 03	0.125/0.016
Clinical strain 10	1/0.25
Clinical strain 14	2/1
Clinical strain 17	0.25/0.031
Clinical strain 49	0.125/0.125
Clinical strain 63	1/0.5
Clinical strain 75	0.25/0.063
Clinical strain 79	0.016/0.016
Clinical strain 80	0.031/0.031
*Staphylococcus aureus*	
ATCC25923	0.125/0.031
Clinical strain 02	0.063/0.016
Clinical strain 14	2/0.25
Clinical strain 19	0.125/0.063
Clinical strain 21	32/4
Clinical strain 24	4/2
Clinical strain 27	2/1
Clinical strain 31	8/4
Clinical strain 33	4/1
Clinical strain 34	4/2
*Klebsiella pneumoniae*	
ATCC10031	1/0.5
Clinical strain 07	0.063/0.031
Clinical strain 18	1/0.25
Clinical strain 20	32/8
Clinical strain 28	0.125/0.016
Clinical strain 39	0.031/0.016
Clinical strain47	0.5/0.063
Clinical strain 64	1/0.125
Clinical strain 70	2/0.5
Clinical strain 73	0.063/0.016

### 2.2. Acute Toxicity Measurement of Tebipenem Pivoxil Tablet in Mice

Within the 14-day observation period, only one mouse was dead in the maximum oral dosage (4.00 g/kg) tebipenem pivoxil tablet group. Thus, the acute toxicity results showed that the minimal lethal dosage (MLD) of the tebipenem pivoxil tablet was 4.00 g/kg and the maximum tolerance dosage (MTD) in the mice was 3.40 g/kg. At 4 h post administration of tebipenem pivoxil tablets, the majority of the animals were prostrate and the activity decreased significantly. At 12 h after the administration, most of the animals were recovered with normal activity, diet, and drinking. There was no significant difference in weight between the tebipenem pivoxil-treated groups and the control group within 14 days (Table 4). The autopsy result showed that the color of the liver and kidney became shallow in some mice, suggesting liver and kidney damage. This damage was dose-dependent (Table 5). Based on the findings above, we speculate that the central nervous system (CNS), liver, and kidney were involved in the acute toxicity caused by the tebipenem pivoxil tablet.

**Table 4:** Body weight of mice in groups administrations within 14 days (g) ($\bar{x}$ ± s, *n* = 20).

Group	Dosage (g/kg)	Body Weight (g)			
		Time after Administration (Day)			
		0	3	7	14
Vehicle	-	19.5 ± 1.6	23.4 ± 1.5	25.2 ± 2.9	26.9 ± 4.0
Tebipenem pivoxil	4.00	18.7 ± 0.7	22.3 ± 1.4	25.4 ± 1.8	27.6 ± 3.2
	3.40	19.5 ± 1.5	23.9 ± 2.6	25.3 ± 3.1	26.3 ± 3.5
	2.89	19.2 ± 1.1	23.2 ± 2.0	25.3 ± 2.7	26.6 ± 2.7

**Table 5:** Incidence of toxicity in liver and kidney after oral administration of tebipenem pivoxil.

Reagent	Dosage (g/kg)	Liver Toxicity		Kidney Toxicity	
		Present	Absent	Present	Absent
Tebipenem pivoxil	4.00	6	13	5	14
	3.20	4	16	4	16
	2.89	3	17	2	18
Vehicle	-	0	0	0	0

### 2.3. Tebipenem Pivoxil Tablet Protects Sepsis Mice from Lethal Challenges with Pathogenic Bacteria

**Figure 3:**
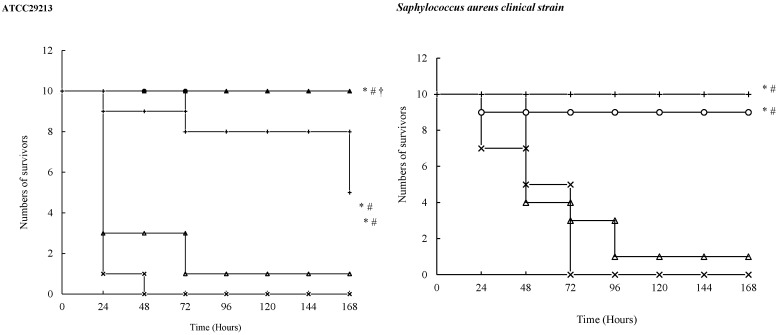

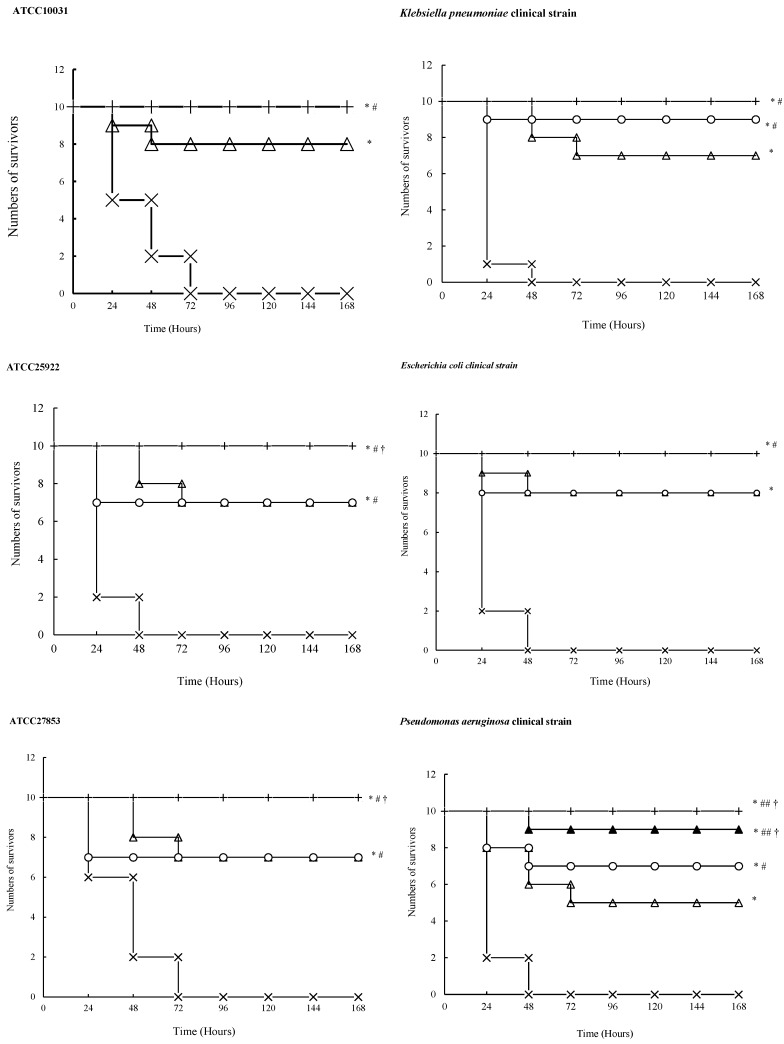
The survival cures for sepsis mice challenged with pathogenic bacteria. After the bacteria injections, the mice received different treatments once. The death of the animals was recorded every day. On the 7th day, the survival curves were made to assess the anti-infection effects of the drugs: Symbols: ×, Vehicle; △, Meropenem 50 mg/kg; ○, Tebipenem pivoxil granules 100 mg/kg; ▲, Tebipenem pivoxil tablet 100 mg/kg; +, Tebipenem pivoxil tablet 50 mg/kg. * *p* < 0.01 *vs.* Vehicle, ^#^ *p* < 0.05, ^##^ *p* < 0.01 *vs.* Meropenem, ^†^ *p* < 0.05 *vs.* Tebipenem pivoxil granules (Kaplan-Meier).

In all the sepsis models, the animals in the vehicle group were dead within 72 h, suggesting successful establishment of the sepsis models. In all the sepsis models, compared with the vehicle, tebipenem pivoxil tablet (50, 100 mg/kg) significantly increased the survival number of the sepsis mice within a 168-h observation period (Figure 3). Further, the survival number in the tebipenem pivoxil tablet group (100 mg/kg) was markedly higher than that in the meropenem group in all the sepsis models tested. In the sepsis models challenged with *Staphylococcus aureus* ATCC29213, *Escherichia coli* ATCC25922, *Pseudomonas aeruginosa* ATCC27853, *Pseudomonas aeruginosa* clinical strain, respectively, tebipenem pivoxil tablet (100 mg/kg) displayed a better protective effect than tebipenem pivoxil granules (100 mg/kg) (Figure 3).

### 2.4. Measurements of ED_50s_ of Tebipenem Pivoxil Tablet in Sepsis Mouse Models

The results revealed that the ED_50s_ of tebipenem pivoxil tablets were (in mg/kg) 7.0281 (95%CI: 5.2918~10.8285), 18.6414 (95%CI: 12.1759~28.5401), 5.3265 (95%CI: 3.6184~7.8444), 2.3205 (95%CI: 1.6219~3.3201), 1.5529 (95%CI: 1.0720~2.2496), 3.0932 (95%CI: 2.1156~4.5224), 20.8060 (95%CI: 14.7041~29.4400), and 20.6961 (95%CI: 14.3390~29.8717) in the sepsis mouse models challenged with *Staphylococcus aureus* ATCC29213, *Staphylococcus aureus* clinical strain, *Klebsiella pneumoniae* ATCC10031, *Klebsiella pneumoniae* clinical strain, *Escherichia coli* ATCC29522, *Escherichia coli* clinical strain, *Pseudomonas aeruginosa* ATCC27853, and *Pseudomonas aeruginosa* clinical strain (Table 6), respectively.

**Table 6:** ED_50s_ of tebipenem pivoxil in various sepsis models.

Strain	Dose (mg/kg)	Survival Number	ED_50_ (mg/kg)	95% CI (mg/kg)
ATCC29213	50.0	10	7.0281	4.8242~10.2389
	25.0	9		
	12.5	7		
	6.25	7		
	3.13	1		
*Staphylococcus aureus* clinical strain	100.0	10	18.6414	12.1759~28.5401
	50.0	7		
	25.0	6		
	12.5	4		
	6.25	2		
	3.13	0		
ATCC10031	25	10	5.3265	3.6176~7.8426
	12.5	8		
	6.25	4		
	3.125	3		
	1.56	1		
	0.78	0		
*Klebsiella pneumoniae* clinical strain	25	10	2.3205	1.6219~3.3201
	12.5	9		
	6.25	9		
	3.125	7		
	1.56	7		
	0.78	0		
ATCC29522	6.25	10	1.5529	1.0720~2.2496
	3.125	8		
	1.56	5		
	0.78	2		
	0.39	0		
*Escherichia coli* clinical strain	12.5	10	3.0932	2.1156~4.5224
	6.25	8		
	3.125	4		
	1.56	3		
	0.78	0		
ATCC27853	100	10	20.8060	14.7041~29.4400
	50	9		
	25	7		
	12.5	3		
	6.25	0		
*Pseudomonas aeruginosa* clinical strain	100	10	20.6961	14.3390~29.8717
	50	9		
	25	6		
	12.5	4		
	6.25	0		

These findings suggested that tebipenem pivoxil tablet had an excellent antibacterial activity against various common pathogenic bacteria, and that tebipenem pivoxil tablet had a stronger anti-infective effect against *Escherichia coli* than other pathogenic bacteria.

## 3. Discussion

To the best of our knowledge, it is first time the *in vitro* and *in vivo* antibacterial properties of tebipenem pivoxil has been systemically investigated. In addition, the preliminary safety of this carbapenem compound was also evaluated.

Previously, in clinical studies in Japan tebipenem pivoxil was usually used for the therapy of infections such as pneumonia [5,7,8,9], acute otitis media [3], or otolaryngological infection [4,9,10]. *Staphylococcus aureus*, *Escherichia coli*, *Klebsiella pneumoniae*, *Pseudomonas aeruginosa* are key pathogenic bacteria causing infections. Particularly for MRSA, the detection rate has been increasing year by year in recent years. MRSA is not only resistant to β-lactam antibiotics such as penicillin, cephalosporin, and enzyme inhibitors containing antibiotics, but also to chloramphenicol, lincomycin, amino-glycosides, quinolones, thus showing multi-drug resistant characteristics [11,12,13]. Actually, infections caused by *Saphylococcus aureus* are often accompanied by other Gram-negative bacteria such as *Pseudomonas aeruginosa*, *Klebsiella pneumoniae* which play a synergistic role in the process of pathopoiesis [6].

Currently, glycopeptide antibiotics represented by vancomycin are the final line of defense against bacterial infections. Along with the increasing amounts and frequency of use of these antibiotics, antibiotic selective pressure is generated and is increasing gradually. Many cases of vancomycin-resistant *Staphylococcus aureus* (VRSA) are now being reported around the world [14,15,16], which makes it more difficult to treat infections caused by MRSA. Besides MRSA, more and more attention is being paid to the infections caused by *Escherichia coli* that carrying some lethal virulence factors [17,18].

Tebipenem pivoxil has an excellent antibacterial ability against most of common pathogenic bacteria, including *Staphylococcus aureus* [1,2], *Escherichia coli* [19], *Klebsiella pneumonia* [5,7,8,9], and so on. In the present study, the MIC values of tebipenem pivoxil against a variety of pathogenic bacteria including 120 kinds of positive Gram-positive bacteria and 380 kinds of Gram-negative bacteria were determined. The results demonstrated that tebipenem pivoxil had an excellent antibacterial effect against most of the bacteria tested and significantly inhibited the growth of the bacteria at or above MIC values, which was similar to results reported in the previous studies [19,20]. In the subsequent *in vivo* experiments, tebipenem pivoxil was applied in the treatment of sepsis caused by pathogenic bacteria.

Sepsis, a systemic inflammatory response syndrome (SIRS) involved in a variety of inflammatory factors caused by infection, is a common complication of severe burns, trauma, and infections. Generally, out of control of sepsis is due to an excessive inflammatory response, but not the direct effect of bacteria or toxins [21]. The occurrence and development of sepsis is not dependent on the persistence of bacteria and toxins. In the event, bacteria and toxins only trigger the initiation of sepsis and the development and severity is actually dependent on physical reactions [22,23].

In the early stage of sepsis, the reasonable use of antibiotics is crucial. In the late stages of sepsis, a large number of Gram-positive bacterial DNA or lipopolysaccharide (LPS) in the dead bacteria are released into the blood after excessive use of antibiotics, causing a febrile reaction, thereby injuring the liver directly or indirectly [22,24]. Any glucose metabolism disorder and changes in enzymes and protein metabolism will lead to activation of coagulation, and the fibrinolytic system, resulting in disseminated intravascular coagulation, gastrointestinal bleeding, and cardiac, renal, pulmonary failure. In addition, bacterial DNA and LPS can activate mononuclear phagocytic cells through a series of intracellular signal transduction, activating NF-κB and rapidly inducing synthesis of TNF-α, IL-6 mRNA and releasing a large number cytokines and inflammatory mediators such as TNF-α and IL-6, eventually mediating the occurrence of sepsis [25,26,27].

## 4. Materials and Methods

### 4.1. Bacterial Strains

One hundred and twenty (120) Gram-positive bacteria, including 20 methicillin-resistant *Staphylococcus epidermidis* (MRSE), 20 methicillin-sensitive *Staphylococcus epidermidis* (MSSE), 20 methicillin-resistant *Staphylococcus aureus* (MRSA), 20 methicillin-sensitive *Staphylococcus aureus* (MSSA), 20 *Pyogenic streptococcus*, 10 *Entercoccus faecalis*, 10 *Enterococcus faecium* and 380 gram-positive bacteria including 85 *Escherichia coli*, 80 *Klebsiella pneumoniae*, 50 *Enterobacter cloacae*, 15 *Proteus mirabilis*, 30 *Enterobacter aerogenes*, 15 *Haemophilus influenzae*, 25 *Citrobacter freundii*, 20 *Serratia marcescens*, 25 *Pseudomonas aeruginosa*, 15 *Stenotrophomonas maltophilia*, and 20 *Acinetobacter baumannii* were clinical isolates collected from hospitals in Beijing region during 2013–2014. Quality control bacteria such as *Staphylococcus aureus* ATCC25923, ATCC29213, *Escherichia coli* ATCC25922, ATCC35218, *Pseudomonas aeruginosa* ATCC27853, *Klebsiella pneumoniae* ATCC 10031, *Entercoccus faecalis* ATCC29212 were purchased from American Type Culture Collection center (ATCC, Manassas, VA, USA). β-Lactams in all the strains were determined by using the filter paper method and validated by the instructions of the American Clinical and Laboratory Standards Institute (CLSI, Wayne, PA, USA).

### 4.2. Experimental Animals

Specific pathogen free (SPF) ICR or KM mice weighing 18–22 g with an equal sex ratio (1:1) were provided by Changsheng Bio-Technology Limited Company (Benxi, China). The animals were given free access to food and water. All the experiments were conducted in accordance with the national guidelines for the care and use of laboratory animals. This study was approved by the Ethnic Committee of Yunnan Institute of Materia Medica (Kunming, China).

### 4.3. Main Reagents

Tebipenem pivoxil tablet (Lot: 20150501) and crude powers (Lot: 20110801) were provided by Yunnan Baiyao Group (Kunming, China). Tebipenem pivoxil granules (Lot: ORPS1113) was purchased from Meiji Pharmaceutic Inc. (Tokyo, Japan). Injectable meropenem (Lot: 110620901) was obtained from United Laboratories (Zhuhai, China). Injectable imipenem and cilastatin sodium (Lot: 121014) was a product of Merck Sharp & Dohme, Hangzhou, China). Injectable ceftriaxone sodium (Lot: b110337415) was provided by Harbin Pharmaceutical Group Co., Ltd. (Harbin, China).

### 4.4. Drug Susceptibility Assay

Cells in log phase (1 × 10^5^ CFU/mL) were cultivated in 96-well plates. The minimal inhibitory concentrations (MICs) were determined by serial two-fold dilutions in MH or LB broth containing various agents in accordance with National Committee for Clinical Laboratory Standards 2006 (NCCLS2006). After that, 100 μL of solution without visible bacteria were uniformly spread on MH or LB agar plates (agent-free) and culture at 35 °C or 37 °C for another 18–24 h. The reagents’ minimal concentrations are thought to be the MBCs when the number of colony forming unit (CFU) is less than 5 on the agar plates.

### 4.5. Acute Toxicity Measurement of Tebipenem Pivoxil Tablet in Mice

After fasting for 12 h, 80 KM mice weighing 18–22 g with an equal sex ratio (1:1) were randomly divided into four groups including three tebipenem pivoxil tablet groups and a vehicle group (20 per group). The animals in the three tebipenem pivoxil groups were respectively orally administrated with different dosages of tebipenem pivoxil tablet (4.00, 3.40, and 2.89 g/kg) according to the body weight once. The mice in the vehicle group were given an equal volume of carboxymethylcellulose sodium once. After the administrations, the general status and death of the mice were recorded every day. The body weight was respectively weighed 0, 3, 7, 14 day post the administrations. At the end of the experiment, the animals were sacrificed and the main organs including heart, liver, spleen, lung and kidney were collected and observed by naked eyes.

### 4.6. Establishment of Sepsis Mouse Models Challenged with Pathogenic Bacteria and Treatments

The ICR mice were randomly divided into five groups including a vehicle group, a meropenem group (50 mg/kg), a tebipenem pivoxil granules group (100 mg/kg), a tebipenem pivoxil tablet low-dose group (50 mg/kg), and a tebipenem pivoxil tablet high-dose group (100 mg/kg). Each group of ten animals included five females and five males.

Active pathogenic bacteria (100% MLD) including *Staphylococcus aureus* ATCC29213 (3.3 × 10^10^ CFU/kg), *Staphylococcus aureus* clinical strain (3.0 × 10^10^ CFU/kg), *Escherichia coli* ATCC25922 (1.5 × 10^10^ CFU/kg), *Escherichia coli* clinical strain (1.25 × 10^10^ CFU/kg), *Klebsiella pneumoniae* ATCC10031 (2.0 × 10^10^ CFU/kg), *Klebsiella pneumoniae* clinical strain (1.65 × 10^10^ CFU/kg), *Pseudomonas aeruginosa* (1.6 × 10^10^ CFU/kg), and *Pseudomonas aeruginosa* clinical strain (1.1 × 10^10^ CFU/kg), respectively, were intravenously injected into the mice. Subsequently, the animals received various treatments once. After that, the death of the animals in the groups was recorded every day. In addition, the diet, weight, and mental state of the animals were also observed and recorded. At the end of this part experiment (within one week), survival curves were made to assess the anti-infection effect of the drugs.

### 4.7. Measurements of 50% Effective Dosages (ED_50_) of Tebipenem Pivoxil Tablet in Bacterial Sepsis Mouse Models

The ICR mice were randomly divided into five or six groups. Active pathogenic bacteria (100% MLD) were then respectively injected into the mice via caudal vein as described above. Subsequently, the animals were administrated with different dosages of tebipenem pivoxil tablet. After that, death of the animals in the groups was recorded every day. At the end of this part of the experiments (within one week), the Bliss method was used to calculate the corresponding ED_50_ of tebipenem pivoxil tablet in each sepsis mouse model.

### 4.8. Statistics and Presentation of Data

The SPSS 11.0 statistics software (SPSS Inc., Chicago, IL, USA) was used to analyze the data. The differences in weight between groups were analyzed using *t*-test. The differences in survival rate among groups were performed by using Kaplan-Meier method. A *p* value of less than 0.05 is considered to be significant.

## 5. Conclusions

*In vivo*, the preliminary acute toxicity measurement was carried out in mice. After that, direct bacterial challenge models were then used as they are closer to real clinical states. Interestingly, single simultaneous tebipenem pivoxil tablet administration significantly reduced the mortality of sepsis model mice challenged with various pathogenic bacteria. Like the *in vitro* results, tebipenem pivoxil showed a powerful protection in sepsis mouse models, agreeing with the previous findings to some degree [3,4,5,7,8,9,10,19]. Furthermore, tebipenem pivoxil tablet had a stronger protective effect than meropenem in all the tested sepsis models, perhaps due to its good tissue distribution [4,28,29]. The tebipenem pivoxil tablet was more effective than the tebipenem pivoxil granules for protecting sepsis mice in some sepsis models perhaps for a higher bioavailability of this tablet *in vivo*. Taken together, our findings show the excellent antibacterial properties of tebipenem pivoxil against various pathogenic bacteria *in vitro* and *in vivo*, which provide some experimental evidence for its application to treat sepsis in the clinic.
